# Reviewer acknowledgement 2013

**DOI:** 10.1186/1755-8166-6-9

**Published:** 2013-04-15

**Authors:** Thomas Liehr, Henry Heng, Yuri Yurov

**Affiliations:** 1 Jena University Hospital, Institute of Human Genetics, Jena, Germany; 2 Center for Molecular Medicine and Genetics, Wayne State University School of Medicine, MI, USA; 3 National Research Center of Mental Health, Moscow, Russia

## Abstract

**Contributing reviewers:**

The editors of *Molecular Cytogenetics* would like to thank all our reviewers who have contributed to the journal in volume 5 (2012).

## 

Joo Wook Ahn

United Kingdom

Maher Albitar

United States of America

Michael Alper

United States of America

Sevim Balcı

Turkey

Samarth Bhatt

Germany

Maria Clara Bonaglia

Italy

Lukrecija Brecevic

Croatia

Arthur Brothman

United States of America

Vincenzo Caputo

Italy

Isabel Maria Marques Carreira

Portugal

Patricia Celestino-Soper

United States of America

Sau Cheung

United States of America

Marcelo Cioffi

Brazil

Edivaldo de Oliveira

Brazil

Maria Descartes

United States of America

Jan Diblik

Czech Republic

Andreas Dufke

Germany

Amanda Figueiredo

Brazil

David Francis

Australia

Ad Geurts van Kessel

Netherlands

Guity Ghaffari

United States of America

Gabriele Gillessen-Kaesbach

Germany

Massimo Giovannotti

Italy

Ekaterina Gornung

Italy

Claudio Graziano

Italy

Susan Gross

United States of America

Claudia Haferlach

Germany

Christine Harrison

United Kingdom

Henry Heng

United States of America

Andreas Houben

Germany

Louanne Hudgins

United States of America

Ivan Iourov

Russia

Ho-Young Kang

South Korea

Nam-Soo Kim

South Korea

Elisabeth Klein

Germany

Nadezda Kosyakova

Germany

Dieter Kotzot

Austria

Tomasz Książczyk

Poland

Anna Kulpanovich

Belarus

Thomas Liehr

Germany

Wenjin Liu

United States of America

Luciana Lourenco

Brazil

Emmanouil Manolakos

Greece

Marina Manvelyan

Armenia

Aurelia Meloni-Ehrig

United States of America

Björn Menten

Belgium

Martina Merkas

Croatia

Hasmik Mkrtchyan

Armenia

Yi Ning

United States of America

Francesca Novara

Italy

Eric Pasmant

France

Philippos Patsalis

Cyprus

Greg Peters

Australia

Elena Raimondi

Italy

Laureana Rebordinos

Spain

Gabriela Repetto

Chile

Nikolai Rubtsov

Russia

Aurora Ruiz-Herrera

Spain

Warren Sanger

United States of America

Catherine Sarri

Greece

Scott Sittler

United States of America

Pawel Stankiewicz

United States of America

David Stevenson

United States of America

Jonathan Strefford

United Kingdom

Vladimir Trifonov

Russia

Karen Ventura

Brazil

Amit Verma

United States of America

Joris Vermeesch

Belgium

Angela Vianna-Morgante

Brazil

Svetlana Vorsanova

Russia

Kunbo Wang

China

Anja Weise

Germany

Xiaojian Xu

United States of America

Peng Zhang

Australia

Orsetta Zuffardi

Italy

